# Supplementary figures and images for: Platelet-rich plasma for rotator cuff tendinopathy: A systematic review and meta-analysis
Source: PLoS One. 2021 May 10;16(5):e0251111. doi: 10.1371/journal.pone.0251111 (PMC8109792; doi:10.1371/journal.pone.0251111)

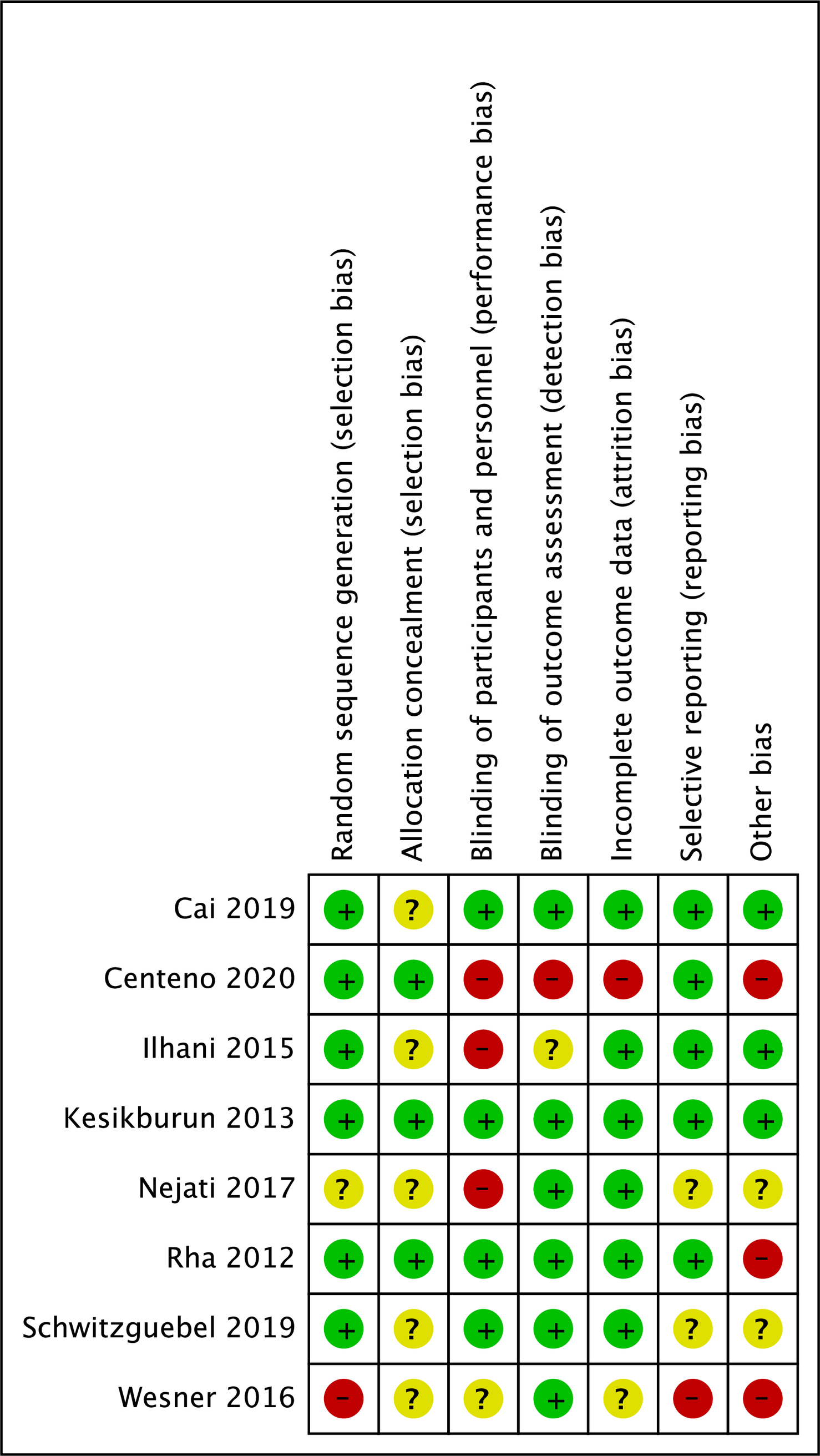

Supplement: S1 Fig — (TIF) [file pone.0251111.s001.tif]

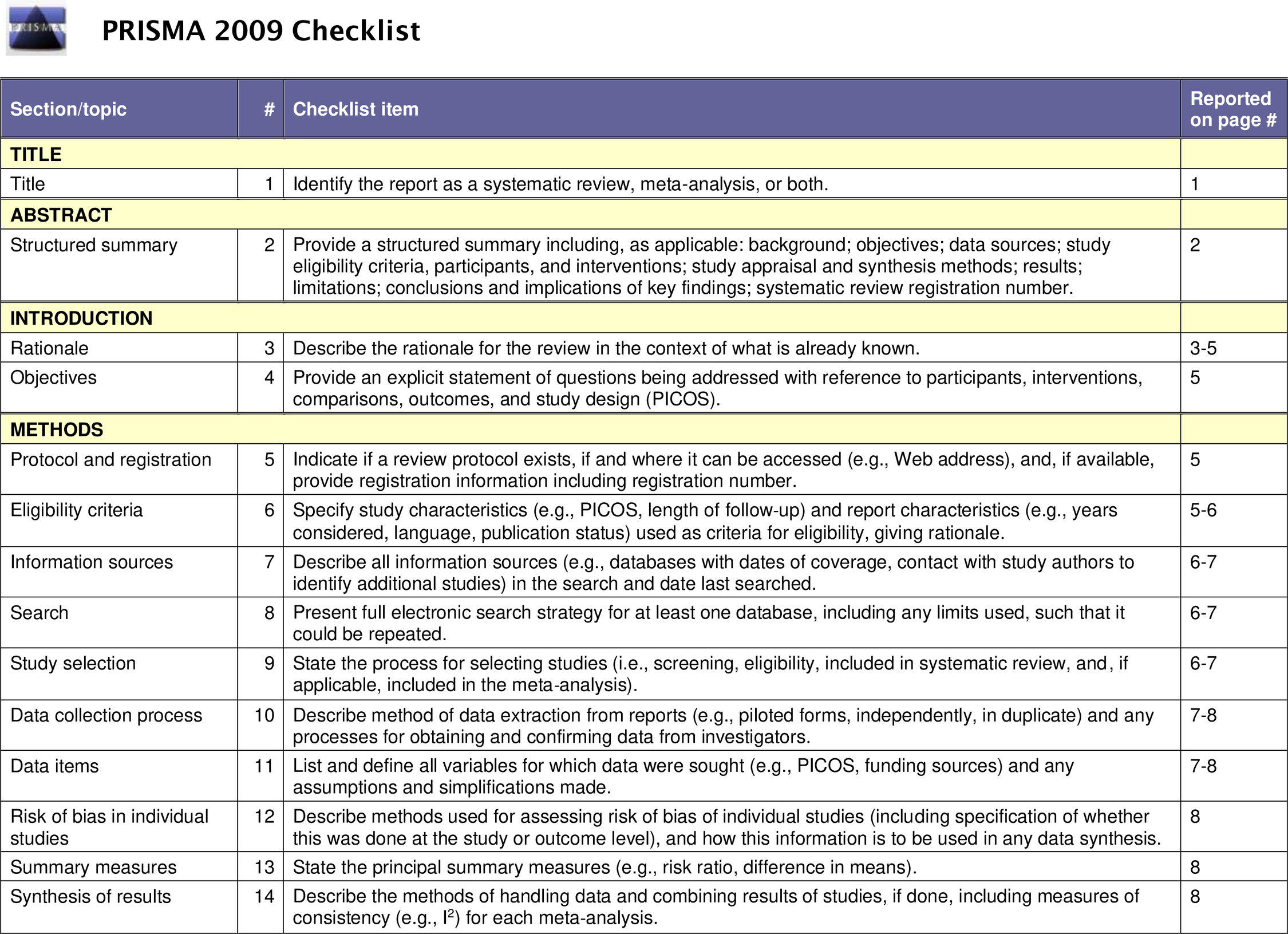

Supplement: S1 File — (TIF) [file pone.0251111.s002.tif]
