# Supplementary material for: Platelet-rich plasma for rotator cuff tendinopathy: A systematic review and meta-analysis
Source: PLoS One. 2021 May 10;16(5):e0251111. doi: 10.1371/journal.pone.0251111 (PMC8109792; doi:10.1371/journal.pone.0251111)
Supplement: S2 File — (DOCX) [file pone.0251111.s003.docx]

**Scopus Search history**

| **No.** | **Search terms** | **No. of documents** |
| --- | --- | --- |
| 1. | ( TITLE-ABS-KEY ( rotator AND cuff ) ) AND ( ( TITLE-ABS-KEY ( platelet-rich AND plasma ) OR TITLE-ABS-KEY ( platelet-rich AND fibrin ) OR TITLE-ABS-KEY ( preparation AND rich AND in AND growth AND factors ) OR TITLE-ABS-KEY ( autologous AND condition AND plasma ) OR TITLE-ABS-KEY ( autologous AND blood ) OR TITLE-ABS-KEY ( platelet AND concentrate ) OR TITLE-ABS-KEY ( platelet AND gel ) OR TITLE-ABS-KEY ( autologous AND growth AND factors ) OR TITLE-ABS-KEY ( platelet AND realasate ) ) ) | 428 |
| 2. | ( ( TITLE-ABS-KEY ( platelet-rich AND plasma ) OR TITLE-ABS-KEY ( platelet AND rich AND fibrin AND matrix ) OR TITLE-ABS-KEY ( autologous AND conditioned AND serum ) OR TITLE-ABS-KEY ( platelet AND concentrate ) OR TITLE-ABS-KEY ( platelet AND gel ) OR TITLE-ABS-KEY ( autologous AND growth AND factors ) OR TITLE-ABS-KEY ( preparation AND rich AND in AND growth AND factors ) OR TITLE-ABS-KEY ( platelet AND releasate )  OR TITLE-ABS-KEY ( platelet AND lysate ) OR TITLE-ABS-KEY ( leucocyte AND platelet AND rich AND plasma ) OR TITLE-ABS-KEY ( platelet AND leucocyte AND rich AND plasma ) ) AND DOCTYPE ( ar ) AND PUBYEAR > 2012 AND PUBYEAR < 2020) AND ( TITLE-ABS-KEY ( muscle AND injury ) AND DOCTYPE ( ar ) AND PUBYEAR > 2012 AND PUBYEAR < 2020) | 274 |
| 3. | ( TITLE-ABS-KEY ( rotator AND cuff ) ) AND ( ( TITLE-ABS-KEY ( platelet-rich AND plasma ) OR TITLE-ABS-KEY ( platelet-rich AND fibbrin ) OR TITLE-ABS-KEY ( preparation AND rich AND in AND growth AND factors ) OR TITLE-ABS-KEY ( autologous AND condition AND plasma ) OR TITLE-ABS-KEY ( autologous AND blood ) OR TITLE-ABS-KEY ( platelet AND concentrate ) OR TITLE-ABS-KEY ( platelet AND gel ) OR TITLE-ABS-KEY ( autologous AND growth AND factors ) OR TITLE-ABS-KEY ( platelet AND realasate ) ) ) | 428 |
